# *Leishmania major* possesses a unique HemG-type protoporphyrinogen IX oxidase

**DOI:** 10.1042/BSR20140081

**Published:** 2014-07-29

**Authors:** Dagmar Zwerschke, Simone Karrie, Dieter Jahn, Martina Jahn

**Affiliations:** *Institute of Microbiology, University Braunschweig, Spielmannstr. 7, D-38106 Braunschweig, Germany; †Cellular and Molecular Neurobiology, Institute of Cell Biology, University Braunschweig, Spielmannstr. 7, D-38106 Braunschweig, Germany

**Keywords:** amastigote partial haem biosynthesis, HemG, *Leishmania major*, parasite, protoporphyrinogen IX oxidase, tetrapyrrole, copro, coproporphyrinogen III, CPO, coproporphyrinogen III oxidase, FeCH, ferrochelatase, IPTG, isopropyl-β-D-thiogalactopyranoside, LB, Luria-Bertani, PPO, protoporphyrinogen IX oxidase, proto, protoporphyrin IX, protogen, protoporphyrinogen IX, TTC*a*, acceptor triphenyltetrazolium chloride

## Abstract

*Leishmania major* was proposed to either utilize haem from its host or partially synthesize the tetrapyrrole from host provided precursors. However, only indirect evidence was available for this partial late haem biosynthetic pathway. Here, we demonstrate that the LMJF_06_1280 gene of *L. major* encodes a HemG-type PPO (protoporphyrinogen IX oxidase) catalysing the oxidation of protoporphyrinogen IX to protoporphyrin IX. Interestingly, trypanosomatids are currently the only known eukaryotes possessing HemG-type enzymes. The LMJF_06_1280 gene forms a potential transcriptional unit with LMJF_06_1270 encoding CPO (coproporphyrinogen III oxidase) and with LMJF_06_1290 for a cytochrome *b*_5_. *In vivo* function of the *L. major hemG* gene was shown by the functional complementation of the *Escherichia coli ΔhemG* strain LG285. Restored haem formation in *E. coli* was observed using HPLC analyses. Purified recombinant *L. major* HemG revealed PPO activity *in vitro* using different ubiquinones and triphenyltetrazolium as electron acceptors. FMN was identified as the *L. major* HemG cofactor. Active site residues were found to be essential for HemG catalysis. These data in combination with the solved crystal structures of *L. major* CPO and the physiological proof of a ferrochelatase activity provide clear-cut evidence for a partial haem biosynthetic pathway in *L. major.*

## Abstract 2

Characterization of the first eukaryotic HemG-type protoporphyrinogen IX oxidase from *Leishmania major*. The cofactor, the electron acceptor and essential active-site amino acid residues were determined for the enzyme. A model for haemoprotein formation for macrophage associated *L. major* was deduced.

## INTRODUCTION

The trypanosomatid of the *Leishmania*-type are flagellated protozoan parasites. These ancient eukaryotes cause Leishmaniasis in 350 million people in 88 countries worldwide with up to 2 million new cases and up to 30000 deaths annually [1]. The disease patterns vary between innocuous cutaneous lesions and lethal visceral forms [1]. Traditional medication is associated with severe adverse effects [1,2]. *Leishmania* spp. have a digenetic life cycle, where they switch between the promastigote life form in the gut of haematophage insect vectors and the amastigote form residing inside phagolysosomes of macrophages [3]. In both forms, they utilize haemoproteins such as cytochrome *a*, *b* and *c* for their electron transport chain-dependent energy conservation [4]. Haem peroxidase, haem-containing protein kinases, flavohaemoglobin, cytochrome *b*_5_ for fatty acid desaturation, enzymes of sulphite oxidation and nitrate reduction as well as multiple cytochrome *P*-450 enzymes constitute further haemoproteins of these organisms [4,5].

**Figure 1:**
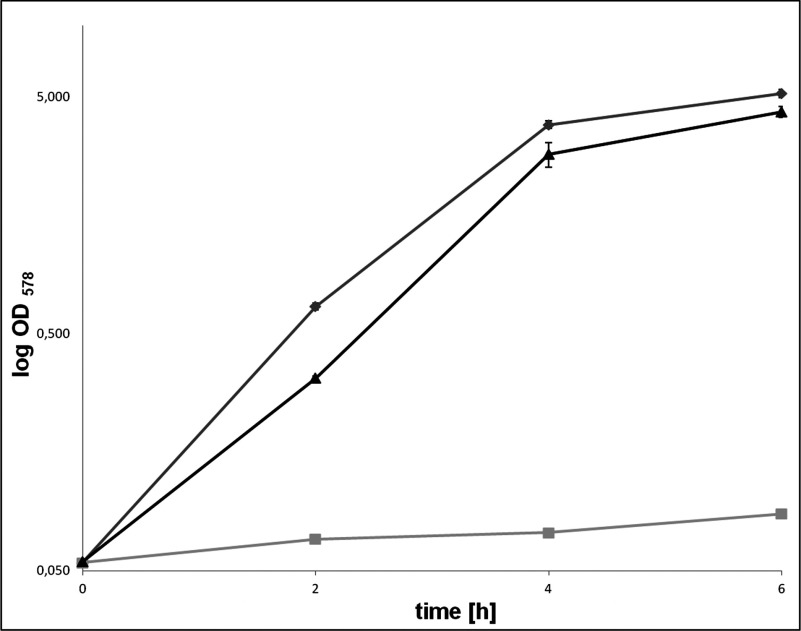
Growth kinetics of complemented *E. coli* ∆*hemG* Complementation of the *E. coli* Δ*hemG* mutant LG285 with *L. major* LMJF_06_1280 cDNA. Cultivation was performed in the LB medium at 37°C and 180 rpm. Optical density at 578 nm was monitored every hour over 6 h. The T-bar indicates the standard deviation *n*=3. *E. coli* mutant LG285 (grey squares); *E. coli* wild-type LE392 (grey diamonds); *E. coli* LG285 pGEXhemGL (black triangles).

In 2012, a *Leishmania* haem uptake transporter for the promastigote state of *Leishmania amazonensis* was described, indicating a direct haem uptake from the gut of the blood-feeding host [6]. Concerning the haem source of the amastigotes only sparse information is available. Early experiments showed that *Leishmania* species were able to grow not only in the presence of haem, but also on defined media supplemented with protoporphyrin IX (proto*a*) [7–9]. This led to the prediction of a functional ferrochelatase (FeCH*a*) [10,11]. Later on, the corresponding gene (LMJF_17_1480) was discovered [12]. Additionally, one potential CPO*a* (coproporphyrinogen III oxidase) LMJF_06_1270) and a potential PPO*a* (protoporphyrinogen IX oxidase) (LMJF_06_1280) were annotated (Figure 1A) [13]. Recently, high-throughput structural biology projects yielded several *Leishmania* spp. CPO crystal structures (PDB # 1VJU, PDB # 2QT8, PDB # 3E8J and PDB # 3EJO, respectively). The overall structure and the bound ligand of the LMJF_06_1270 encoded protein finally identified it as oxygen-dependent CPO (PDB # 3DWR, PDB # 3DWS) [14,15]. Transcriptome analyses identified transcripts from the LMJF_06_1270 and LMJF_06_1280 genes indicating their functional expression [16]. The proposal was that the *Leishmania* spp. may take up copro*a* (coproporphyrinogen III) from their macrophage host which then is converted into haem [4]. However, no experimental evidence was available for the function of the potential PPO protein.

In nature the six electron oxidation of protoporphyrinogen IX (protogen*a*) to the first coloured intermediate of haem biosynthesis proto is catalysed by a set of highly diverse, even partially completely unknown enzymes. Very recently the novel HemJ-type of PPO was discovered in cyanobacteria and *Acinetobacter* spp. [17,18]. Almost all haem synthesizing eukaryotes and some bacteria utilize the oxygen-dependent FAD enzyme of the HemY-type [19]. Database searches revealed that the only exceptions to this rule are trypanosmatid protozoa of the *Leishmania* class, which were proposed to possess a HemG-type PPO.

In addition, only a few bacteria, including several Enterobactericaea employ the flavodoxin-like FMN enzyme HemG [20,21]. The enzyme transfers six abstracted electrons via quinones to various terminal oxidases of the respiratory electron transport chains, which use oxygen or nitrate and fumarate under anaerobic conditions as electron acceptors. This allows the coupling of anaerobic and aerobic haem biosynthesis to cellular respiration. Thus, the reaction contributes to proton gradient formation [21].

Here, we demonstrate for the first time the activity of a eukaryotic HemG-type PPO. Thus, partial haem biosynthesis from phagocyte-derived haem precursors in *L. major* is highly probable. It serves most probably to haemoprotein formation during the amastigotic state in the macrophage [4].

## EXPERIMENTAL

### Bacterial strains and constructed plasmids

The *Leishmania major hemG* cDNA of LMJF_06_1280 optimized for *E. coli* codon usage (http://www.jcat.de) was cloned into the *Bam*HI and *Not*I sites of either the glutathione S-transferase-tag encoding vector pGEX 6P-1 (Amersham Biosciences Little Chalfont/GB) resulting in plasmid pGEXhemGL or in the His-tag encoding vector pET32a (Novagen) resulting in plasmid pET32ahemGL. For complementation studies, pGEXhemGL was transformed into the *E. coli* LG285 Δ*hemG* mutant cell line [*hemG::KmR*, *supE44*, *supF58*, *HsdR514*, *galK2*, *galT22*, *metB1*, *trpR55*, *lacY1*] [22]. *E. coli* LE392 [*glnV44*, *supF58* (*lacY1* or Δ*lacZY*), *galK2*, *galT22*, *metB1*, *trpR55*, *hsdR514* (*rK-mK^+^*)] (Promega) was used in complementation studies as wild-type strain. Cell-free extracts for enzyme activity analyses were prepared from *E. coli* OverExpress™ C43 (DE3) cells (Lucigen) for cells carrying the plasmid pGEXhemGL. *E. coli* BL21-CodonPlus®-RIL (Stratagene) carrying pET32ahemGL was used for protein production for subsequent purification and renaturation. The *HemG* variant genes were generated using the Q5® Site-Directed Mutagenesis Kit (New England Biolabs) according to the manufacturer's instructions. Correctness of introduced mutations in the genes was proven by DNA sequencing (GATC).

### Complementation studies

*E. coli* LG285 Δ*hemG* cells carrying either the pGEXhemGL or pGEX 6P-1 as control were cultivated in 50 ml LB (Luria-Bertani) medium supplemented with 500 μM IPTG*a* (isopropyl-β-D-thiogalactopyranoside) and 0.3 mM ampicillin at 37°C with shaking at 180 rpm. The corresponding wild-type strain *E. coli* LE392 was cultivated in 50 ml LB supplemented with 500 μM IPTG at 37°C at 180 rpm. Samples were taken every 2 h over 10 h and OD (optical density) was determined photometrically by 578 nm (Ultrospec 500 Pro, Amersham Biosciences). HPLC cultures were centrifuged by 2500×***g***, and cells were mechanically disrupted with glass beads (100 μm) in 50 mM Tris–HCl pH 8.0 containing 2% (v/v) Tween 80 using FastPrep®-24 Instrument (MP Biomedicals). Tetrapyrroles were extracted from cell-free extracts with a HCl/acetone (2.5:97.5) solution as described before [23]. Isolated tetrapyrroles were separated by reversed phase chromatography using an HPLC-system 2000 series (Jasco) and an Equisil BDS-C18 reversed phase column (Dr Maisch, Ammerbuch-Entringen, Germany) using a modified method of Lim [23].

### Production of cell-free *E. coli* extracts harbouring *L. major* HemG

A 50 ml culture of *E. coli* OverExpress^(tm)^ C43 (DE3) carrying pGEXhemGL was grown in LB medium supplemented with 0.3 mM ampicillin at 37°C with shaking at 180 rpm. When the culture reached an OD_578_ of 0.9, protein production was induced with the addition of 500 μM IPTG. The cells were further cultivated at 25°C and 180 rpm for 4 h, harvested and disrupted as described above. The suspension was centrifuged for 1 h by 2500×***g*** and the supernatant was used as cell-free extract for PPO activity assays.

### Production and purification of *L. major* HemG

Two litres of *E. coli* BL21-CodonPlus®-RIL cells carrying pET32ahemGL were grown in LB medium supplemented with 0.3 mM ampicillin at 37°C with shaking at 180 rpm. When the culture reached an OD_578_ of 0.6, recombinant protein production was induced by the addition of 500 μM IPTG. The *E. coli* cells were further cultivated at 37°C, with shaking at 180 rpm for 3.5 h and subsequently harvested. For isolation of the HemG-containing inclusion bodies the cell pellet was redissolved in 10 ml harvesting buffer 100 mM Tris–HCl, pH 8.0, lysozyme 0.2 mg/ml incubated for 5 min at room temperature and disrupted by sonication (0.5 s pulse, 0.5 s pause, 70% amplitude; KE76; Sonoplus HD 2070). The resulting cell-free suspension was centrifuged (125000×***g***, 4°C) and the resulting pellet was washed twice in 6 ml isolation buffer 2 M urea, 20 mM Tris–HCl, pH 8.0, 500 mM NaCl, 0.2% (w/v) Triton X-100. The proteins were solubilized by sonication (0.5 s pulse, 0.5 s pause, 70% amplitude; KE76; Sonoplus HD 2070) and residual debris was removed by centrifugation (125000×***g*** at 4°C). Subsequently, the pellet was washed in 7 ml 100 mM Tris–HCl (pH 8.0). The purified inclusion bodies were redissolved in 10 ml buffer S containing 6 M guanidinium–HCl, 20 mM Tris–HCl (pH 8.0), 500 mM NaCl, 5 mM β-mercaptoethanol [24] and dialysed against buffer S without guanidinium–HCl twice for 24 h.

### PPO activity assays

Approximately 2 mg of protoporphyrin IX (Sigma-Aldrich) were dissolved in 7 ml of 10 mM KOH in 20% (v/v) ethanol and stirred 20 min in the dark. From this stock solution 2 ml were diluted into 3 ml of 10 mM KOH in 20% ethanol to create the working solution. The concentration of the working solution was determined by diluting 30 μl of it with 3 ml of 2.7 M HCl and measuring the absorbance at 408 nm, using the millimolar extinction coefficient value of 297 cm/mM (Ultrospec 500 Pro, Amersham Biosciences). Three millilitres of the working solution were reduced using 6 g freshly prepared pulverized 3% (w/v) sodium mercury amalgam under nitrogen atmosphere [25]. The colourless reduction product protogen was filtered and used for the activity assay. Assay conditions were used as previously reported [21] with 0.62 mg/ml cell-free extract or 0.4 mg/ml pure protein.

### Determination of the HemG flavin cofactor

For the determination of the HemG flavin cofactor recombinantly produced and purified protein was precipitated using 5% (v/v) perchloric acid and centrifuged at 10000×***g*** at room temperature for 10 min. The supernatant was analysed on an ODS Hypersil 250×4.6 mm column (Techlab) using a HPLC-system 1500 series (Jasco Gross-Umstadt). An isocratic gradient was employed with acetonitrile/water/triflouric acid/phosphoric acid (75%) (14/84.41/1.5/0.09) as the mobile phase and a flow rate of 0.5 ml/min at 30°C. Cofactor detection was performed by measuring the fluorescence using an excitation wavelength of 430 nm and an emission wavelength of 525 nm. Photometric diode array analysis from 200 to 650 nm wavelength was performed simultaneously [21].

## RESULTS AND DISCUSSION

### The *L. major* LMJF_06_1280 encoded protein reveals high amino acid sequence similarity to the HemG-type PPO

In order to answer the question for the existence of a partial haem biosynthetic pathway in *L. major* during its amastigotic, macrophage-associated life stage, the function of the LMJF_06_1280 gene encoded potential PPO was analysed. The amino acid sequence deduced from the corresponding cDNA showed approximately 50% amino acid sequence identity to the HemG-type PPO of *E. coli*. Interestingly, *L. major* HemF-type CPO (LMJF_06_1270) and FeCH (LMJF_17_1470) displayed 64 and 53% amino acid sequence identity to their *E. coli* counterparts, respectively [13]. These high amino acid sequence homologies are astounding because until now only Betaproteobacteria where found to be symbionts in the family of kinetoplastids [13]. Thus, horizontal gene transfer from Gammaproteobacteria might provide an explanation. In addition to Gammaproteobacteria, HemG-type PPOs*a* were also found in selected species of Alphaproteobacteria (*Pseudovibrio*), Betaproteobacteria (*Thauera*), Cyanobacteria (*Prochlorococcus*) and even Archaea (*Halograneum*). The principle of this unique distribution and its underlying selection pressure remains to be elucidated.

### *L. major* HemG serves as PPO in *E. coli* haem biosynthesis

In order to test for the PPO function of the *L. major* LMJF_06_1280 encoded HemG *in vivo*, the haem auxotrophic *E. coli* Δ*hemG* mutant LG285 strain [22] was complemented with the corresponding *L. major* cDNA synthezised in *E. coli* codon usage and cloned into an appropriate vector. Growth experiments of the wild-type *E. coli* strain LE392, the Δ*hemG* mutant LG285 and the complemented *hemG* mutant strains showed wild-type-like growth of the complemented *E. coli* Δ*hemG* mutant, whereas the not complemented mutant strain LG285 almost failed to grow because of its haem auxotrophy (Figure 1). In detail, the growth rates of the corresponding *E. coli* wild-type strain LE392 and the complemented *E. coli* Δ*hemG* strain were approximately the same (*k*=2.281 and 2.186, respectively). The non-complemented mutant showed a much weaker growth (*k*=0.238) (Figure 1) and the reddish colour of the culture indicated an accumulation of haem precursor molecules as described earlier [26].

**Figure 2:**
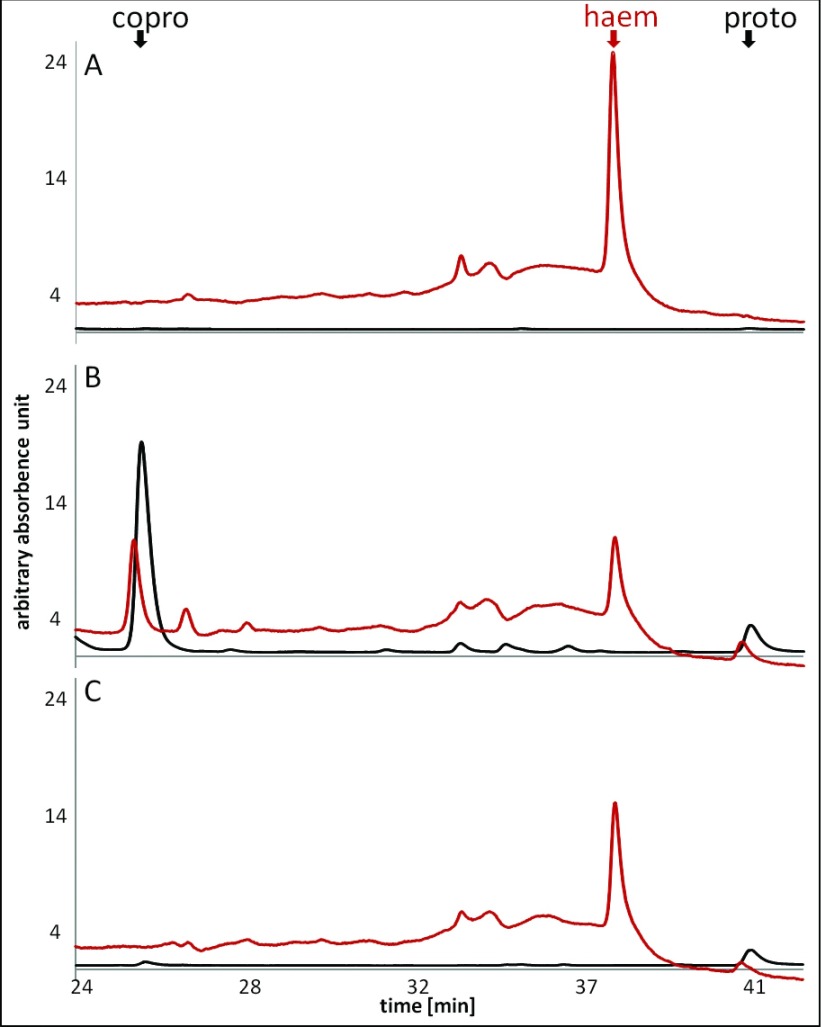
Restored haem biosynthesis in *E. coli* LG285 when complemented with *L. major* LMJF_06_1280 cDNA HPLC analyses using a reversed phase column for the separation and identification of haem, proto and copro were performed. UV/Vis spectra (red) and fluorescence spectra at 409 nm (black) were recorded simultaneously. The retention time for haem was 37.3 min., for proto 40.7 min. and 25.6 min. for copro. (**A**) *E. coli* wild-type LE392; (**B**) *E. coli* Δ*hemG* mutant LG285; (**C**) *E. coli* Δ*hemG* mutant LG285-pGEXhemGL.

In order to proof that the observed growth of the *E. coli* Δ*hemG* mutant complemented with the *L. major* LMJF_06_1280 cDNA was due to restored haem biosynthesis, HPLC analyses for haem and its biosynthetic precursors were performed. For this purpose, tetrapyrroles were extracted from the various *E. coli* strains and separated using reversed phase chromatography as described in the Experimental section. UV/Vis spectra were recorded for the identification of haem and fluorescence spectra for the identification of the porphyrins. During tetrapyrrole extraction the unstable porphyrinogens get converted into porphyrins. Consequently, protogen and proto cannot be distinguished by this method. As expected, in wild-type *E. coli* LE392 only haem and no biosynthetic intermediates were identified (Figure 2A). In contrast in the mutant strain LG285, the haem precursors proto/protogen, and mainly coprogen*a* were found (Figure 2B). The low amount of haem in the Δ*hemG* background is most likely derived from the chemical interconversion of protogen into proto with the subsequent enzymatic iron insertion. However, this chemical process allows only for limited growth (see Figure 1). The accumulation of copro was already observed in the 1960s and 1970s after supplementation of bacterial cell cultures with 5-aminolevulinic acid [27], indicating a general limitation at the CPO-catalysed step in haem biosynthesis. In agreement, mutations in the late steps of haem synthesis often result in the accumulation of mainly coprogen in addition to the substrate to be metabolized by the inactivated enzyme [27]. Therefore coprogen accumulation is typical for most mutants of the late haem biosynthetic pathway. In the complemented *E. coli*, Δ*hemG* mutant rescued by the *L. major hemG*, the amount of proto/protogen and copro was found drastically reduced compared with the *ΔhemG* mutant strain, whereas the haem level was found increased (Figure 2C). Together with the growth experiments, these data clearly demonstrate PPO activity for the *L. major* LMJF_06_1280 gene product *in vivo*. The gene and the enzyme will be referred to as *L. major hemG* and *L. major* HemG, respectively.

### *L. major* HemG transfers electrons from protoporphyrinogen IX to the fumarate reductase system of *E. coli*

Subsequently to the confirmation of the *in vivo* activity of *L. major* HemG, classical HemG assays were employed to demonstrate PPO activity *in vitro*. In the late 1970s it was already observed using *E. coli* cell-free extracts, that the tested PPO activity required ubiquinones or the menaquinone containing the respiratory fumarate system as electron acceptors [25]. For cell-free extract preparation the respective *E. coli* cells were grown under anaerobic conditions without nitrate addition in order to solely induce fumarate reductase formation. Under anaerobic growth conditions, fumarate reductase constitutes an alternative electron acceptor system to the oxygen respiratory machinery. Testing for nitrate respiration as electron accepting system for *L. major* HemG served as negative control, since the anaerobic onset for the production of the corresponding enzyme system requires the presence of nitrate in the growth medium [21]. As expected, the *L. major* HemG activity in *E. coli* cell-free extracts was solely seen in the presence of electron accepting ubiquinone-1 or of fumarate allowing electron transfer from protogen to fumarate via menaquinone containing fumarate reductase. Residual background reactivity of ubiquinone with protogen was observed.

**Figure 3:**
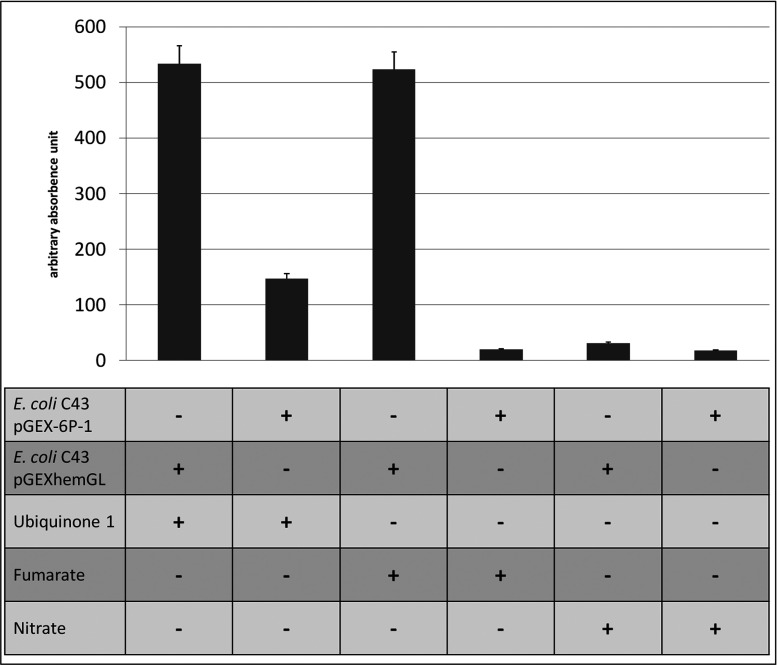
PPO activities of *E. coli* cell-free extract containing *L. major* HemG PPO activities were obtained as described in Experimental. Proto formation was tested with fumarate, nitrate or ubiquinone-1 as electron acceptor. Arbitrary absorbance units related to the relative fluorescence after 60 min of enzyme assay are given. The T-bar indicates the standard deviation for *n*=3.

Thus, a dependency of *L. major* HemG catalysis on respiratory electron transport (Figure 3) was demonstrated. Furthermore, it was shown that ubiquinone-1 can act as direct electron acceptor.

### Purified *L. major* HemG has PPO activity *in vitro*

**Figure 4:**
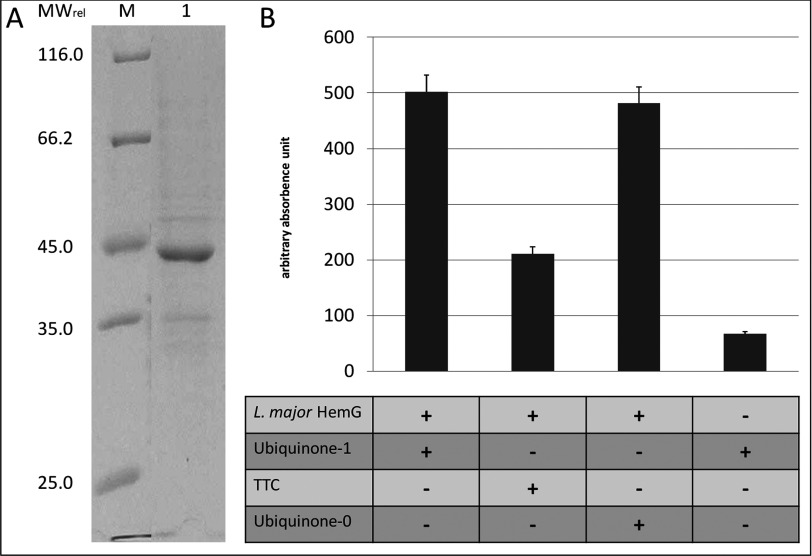
Recombinant production, purification and proto formation of *L. major* HemG (**A**) *L. major* HemG after recombinant production in *E. coli*, purification from inclusion bodies and renaturation to solubility was subjected to SDS–PAGE (12% gel) and visualized by Coomassie Brillant Blue staining. Lane M: molecular weight standard; lane 1: affinity-tagged *L. major* HemG. (**B**) PPO activities of purified *L. major* HemG. PPO activities were obtained as described in Experimental. Proto formation was measured with addition of TTC, ubiquinone-0 or ubiquinone-1 as electron acceptors and purified enzyme as shown in lane 1. Arbitrary absorbance units: relative fluorescence units with *t*=60 min. The T-bar indicates the standard deviation for *n*=3.

To ultimatively demonstrate PPO activity for *L. major* HemG, purified recombinant protein was analysed *in vitro. L. major* HemG fused to a His-tag was produced in *E. coli*. The protein found misfolded in inclusion bodies was isolated, denatured and refolded, yielding up to 2 mg/l apparently homogeneous protein. A protein with a *M*_r_ of 42.000 (± 5000) was observed on SDS–PAGE. This is in good agreement with the calculated molecular mass deduced from the amino acid sequence of the *L. major* HemG fusion protein. Removal of the His-tag did not change the enzymatic behaviour, consequently further assays were performed with the fusion proteins. The purified HemG was tested with two ubiquinones and the artificial electron TTC*a* (triphenyltetrazolium chloride). Under all tested conditions electron transfer from protogen to the electron acceptors with the formation of proto was detected (Figure 4). The observed fluorescence is direct proportional to proto formation. The PPO activity differed for the different tested electron acceptors between 593 nM proto/mg protein/h for ubiquinone-1, 572 nM proto/mg protein/h for ubiquinone-0 and 211 nM proto/mg protein/h for TTC, respectively. Again, residual reactivity of ubiquinone with protogen was observed. Observed enzyme activities for the *L. major* PPO were approximately ten times higher compared with the values obtained for *E. coli* HemG [21]. Thus, *L. major* HemG was finally directly identified as a PPO.

### FMN is the cofactor of *L. major* HemG

**Figure 5:**
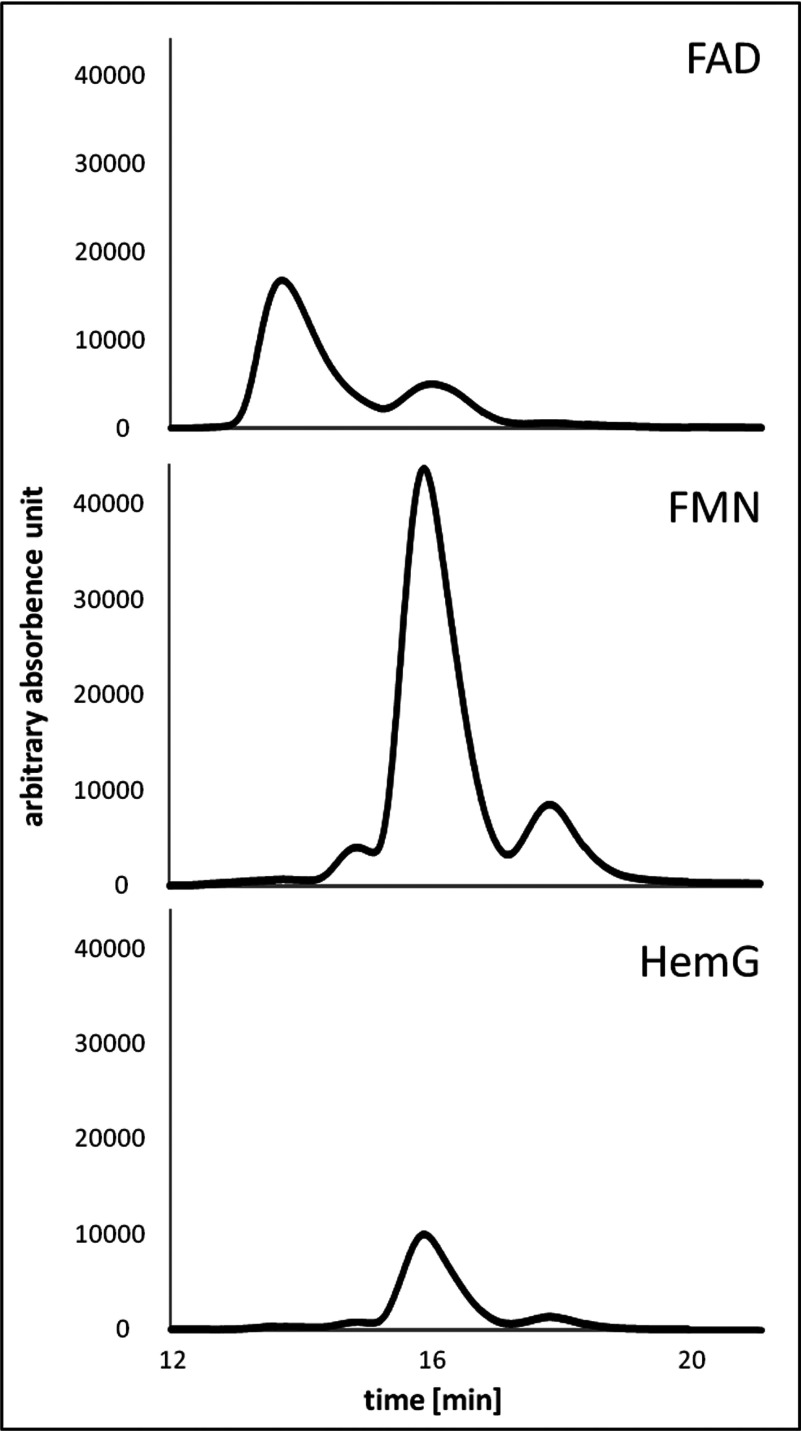
HPLC analysis for the identification of the flavin cofactor of *L. major* HemG The retention time for the cofactor FAD was 13.28 min and 15.55 min for FMN. The purified cofactor from the *L. major* HemG fraction was detected at 15.55 min and thus identified as FMN.

PPO’s*a* of the HemY and the HemG classes are utilizing flavins for the six electron oxidation of protogen. Eukaryotic HemY PPOs*a* possesses FAD as cofactor, whereas *E. coli* HemG is known to employ FMN. In order to reveal the cofactor of *L. major* HemG, the purified protein was denatured using perchloroacetic acid and the resulting supernatant was analysed using HPLC separation. The obtained UV/Vis spectrum of the respective chromatography was compared with the corresponding spectra from commercial standards of FAD and FMN. The retention times of both flavines were used for the identification of the *L. major* cofactor. Figure 5 presents the recorded spectra. The flavin extracted from *L. major* HemG was identified as FMN (Figure 5). The FMN was obviously non-covalently bound, based on the extraction method.

### Analysis of the active site of *L. major* HemG

**Figure 6:**
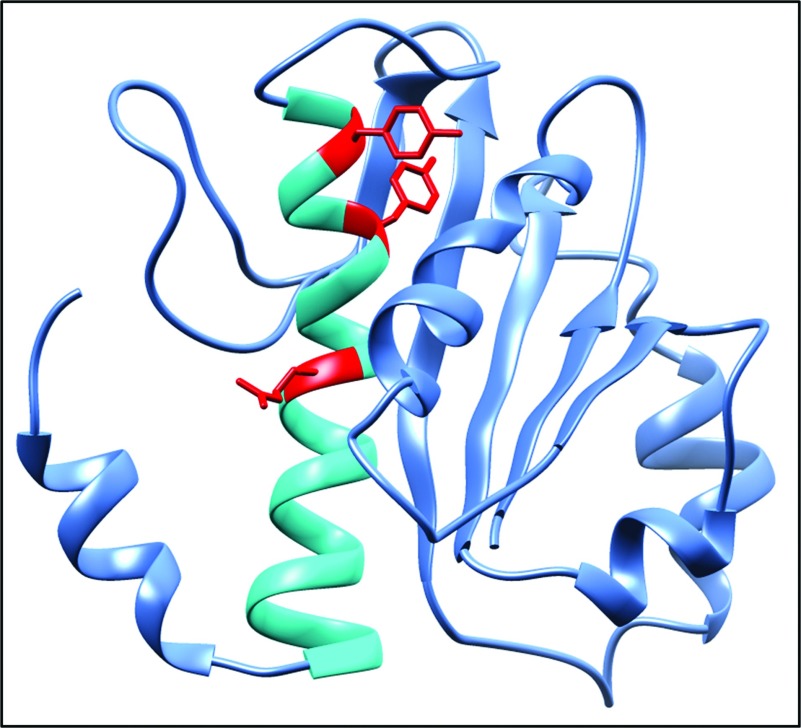
Protein model of *L. major* HemG The HemG model was created by the open access Swiss Model. The predicted active site is displayed in cyan and the locations of the mutated amino acid residues for the HemG variants are indicated in red.

**Figure 7:**
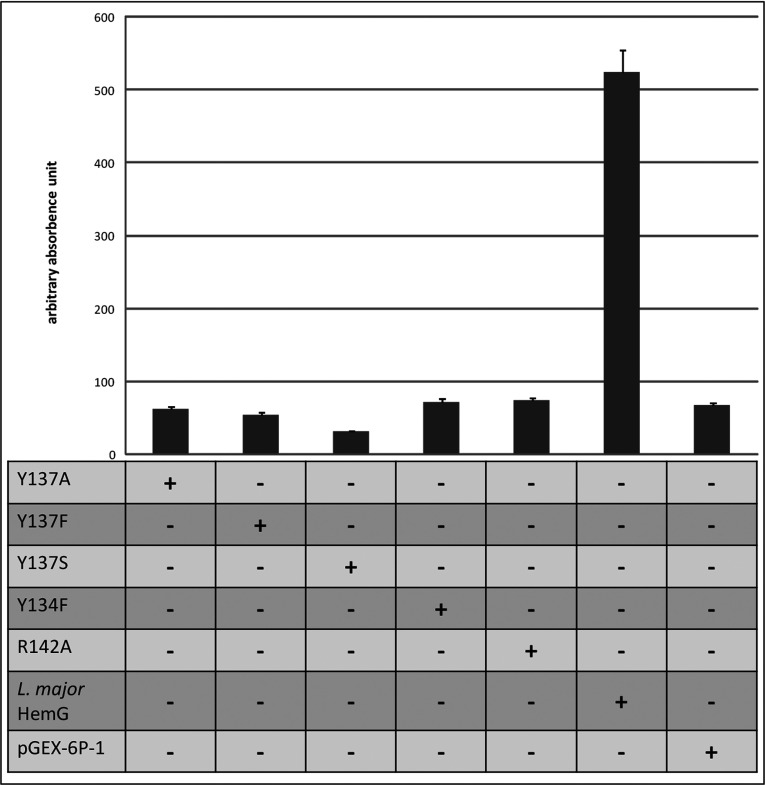
PPO activities of *E. coli* cell-free extract containing *L. major* HemG mutants PPO activities were obtained as described in the Experimental section. Proto formation was depicted with fumarate as electron acceptor. Arbitrary absorbance units: relative fluorescence units with *t*=60 min. The T-bar indicates the standard deviation for *n*=3.

HemG proteins are highly related to flavodoxin proteins [20]. Based on the solved crystal structure of a related flavodoxin the structure of *E. coli* HemG was modelled [20,21]. Boynton et al. proposed a putative active site between amino acid residues 124 and 149 of *E. coli* HemG (Figure 6). This long loop represents an insertion into the flavodoxin backbone. Deletion of this loop of HemG resulted in the inactivation of the enzyme [20]. Here we analysed the corresponded active site of *L. major* HemG. Two tyrosines at positions 134 and 137 as well as the arginine at position 142 are highly conserved among HemG analogues (Figure 6). Tyrosine residues are known for their electron transport capacity through proteins because of their delocalized *π*-electron systems [28]. Arginines are often involved in tetrapyrrole binding via ring substituents coordination [29]. To investigate the contribution of these conserved amino acid residues to the *L. major* HemG activity a site directed mutagenesis approach was pursued. We exchanged tyrosine 134 against phenylalanine leading to the HemG variant Y134F. Tyrosine at position 137 was exchanged against phenylalanine, alanine or serine residues leading to the HemG variant Y137F, Y137A and Y137S, respectively (Figure 6). The arginine at position 142 was mutated to alanine leading to R142A. Phenylalanine differs from tyrosine only by the lack of the hydroxyl group in the ortho position on the benzene ring, Therefore an influence on the protein shape is unlikely. The serine hydroxyl group might be necessary for catalysis. For all variants the PPO activity was examined using cell-free extracts of *E. coli* expressing *L. major* HemG variants as shown in Figure 7. An *E. coli* cell-free extract without *L. major* HemG was used as control. Fumarate was used as electron acceptor (Figure 7).

**Figure 8:**
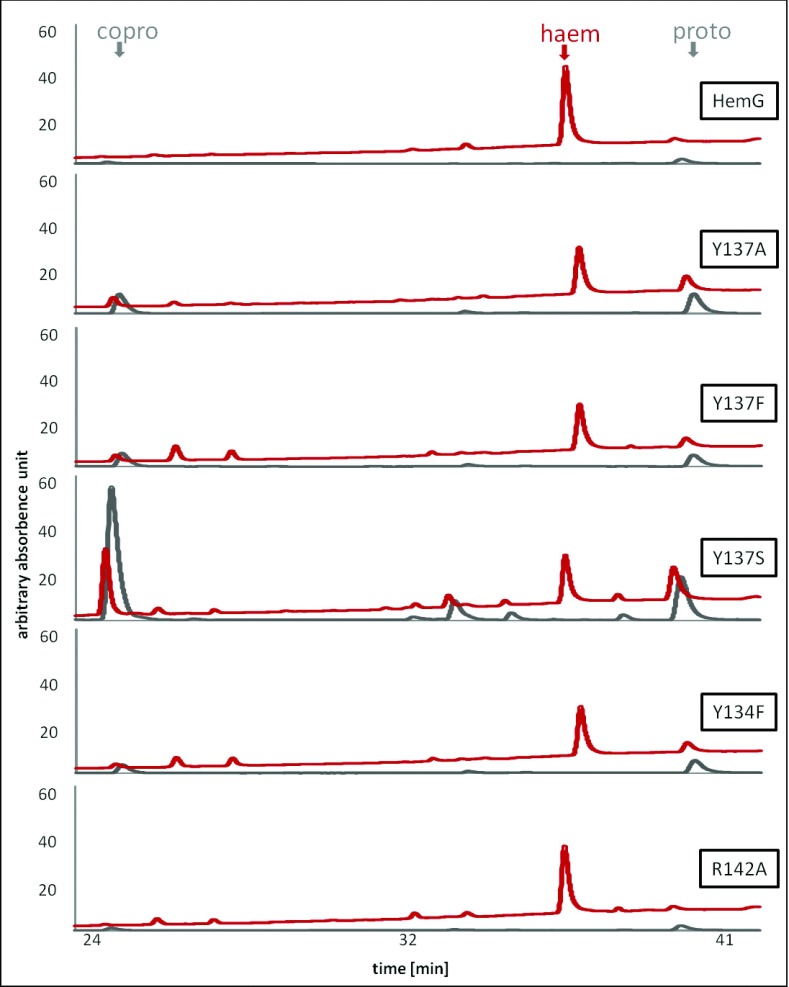
Spectral analysis of the influence of *L. major* HemG mutants on haem biosynthesis Spectral analysis of the influence of *L. major* HemG mutants on haem biosynthesis of mutant LG285 using HPLC analysis for the identification of haem, proto and copro. UV/Vis spectra (red) and fluorescence spectra (grey) at 409 nm were recorded simultaneously. The retention time for haem was 37.3 min, for proto 40.7 and 25.6 min for copro. Assayed HemG variants are indicated.

To further verify these *in vitro* data the *hemG-*deficient strain LG285 was complemented with the various mutated *hemG* genes. Subsequent HPLC analyses for the detection of haem and its precursors were performed. Figure 8 shows HPLC analyses of the five tested *L. major* HemG variants. The grey line re-presents the fluorescence spectra at 409 nm used for the identification of copro and proto. UV/Vis spectra are shown in red revealing the signal for haem.

An activity assay of HemG variant R142A showed a 50 times lower activity compared with the wild-type *L. major* HemG; however, still slight enzyme activity was visible (Figure 7). HPLC analyses revealed almost no precursor molecules but low haem amounts (Figure 8). These results indicate a retarded reaction of the R142A. These results suggest a role of HemG residue R142 in substrate binding. Moreover, the HemG variant Y134F revealed residual PPO activity (Figure 7). In agreement, residual haem formation was observed with the typical accumulation of haem precursor molecules. Obviously, this residue is important but not essential to *L. major* HemG activity. In contrast, amino acid exchanges at position Y137 led to complete inactivation of the enzyme (Figure 7). Similar to the *E. coli* Δ*hemG* mutant control, significant reduction of haem formation with the parallel accumulation of the haem precursors was observed. The strongest phenotype was detected for HemG variant Y137S. Obviously, residue Y137 is essential to *L. major* HemG activity suggesting a crucial role in electron transfer from the substrate.

### Conclusions

**Figure 9:**
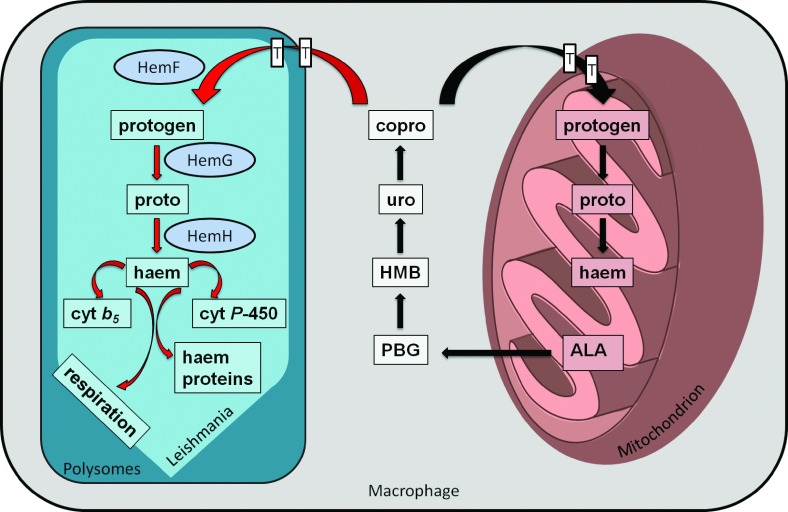
Model for the intracellular localization of haem synthetic enzymes and haem trafficking in *L. major* amastigotes Illustrated is a mammalian macrophage in which the haem biosynthesis starts within the mitochondrion (right side). Haem precursors are then transported into the cytosol (grey) and further processed to copro. Finally, copro is transported via membrane-bound transporters into *L. major* (cyan) located in the polysomes (left side), where partial haem biosynthesis via the activity of the last three enzymes yield in haem for integration into multiple haemoproteins. The abbreviated enzyme names are: HemF, aerobic coproporphyrinogen III oxidase; HemG, FMN-containing protophyrinogen IX oxidase; HemH, FeCH. The abbreviated haem precursor molecules and hemoproteins are: ALA, δ-aminolevulinic acid; PBG, porphobilinogen; HMB, hydroxymethylbilane; uro, uroporphyrinogen III; copro, coproporphyrinogen III; protogen, protoporphyrinogen IX; proto, protoporphyrin IX; cyt *b*_5_, cytochrome *b*_5_; cyt *P*-450*a*, cytochrome *P*-450, respectively.

*L. major* is a dangerous pathogen responsible for the deaths of approximately 30000 people/year. The organism requires haem for its multiple essential haemoproteins. However, *L. major* does not possess a complete haem biosynthesis. Obviously, the promastigote form of the pathogen acquires haem via haem transporter mediated import [6]. Here, PPO activity for the HemG-type *L. major* protein encoded by LMJF_06_1280 was demonstrated using *in vivo* as well as *in vitro* analyses. Structural biology identified the protein encoded by LMJF_06_1270 as CPO (see the Introduction section). Corresponding genes are expressed in *L. major* [16]. Finally, physiological evidence and a potential gene coding for FeCH (LMJF_17_1480) are available [7–9]. Our data suggest that the amastigote form localized in the macrophages utilizes coprogen from the host to produce haem (Figure 9). Corresponding coprogen transporters remain to be identified. Since trypanosomatids are the only eukaryotes possessing a HemG-type PPO, the *L. major* enzyme represents a perfect drug target for the treatment of Leishmaniasis most probably without the detrimental side effects of today's treatment.
